# Controllable deposition of organic metal halide perovskite films with wafer-scale uniformity by single source flash evaporation

**DOI:** 10.1038/s41598-020-75764-5

**Published:** 2020-11-02

**Authors:** Woocheol Lee, Jonghoon Lee, Hyeon-Dong Lee, Junwoo Kim, Heebeom Ahn, Youngrok Kim, Daekyoung Yoo, Jeongjae Lee, Tae-Woo Lee, Keehoon Kang, Takhee Lee

**Affiliations:** 1 Department of Physics and Astronomy, and Institute of Applied Physics, Seoul National University, Seoul, 08826 Korea; 2 Department of Materials Science and Engineering, Seoul National University, Seoul, 08826 Korea; 3 School of Earth and Environmental Sciences, Seoul National University, Seoul, 08826 Korea; 4 School of Chemical and Biological Engineering, Institute of Engineering Research, Research Institute of Advanced Materials, Nano Systems Institute (NSI), Seoul National University, Seoul, 08826 Korea

**Keywords:** Materials for devices, Materials for optics, Optical materials and structures

## Abstract

Conventional solution-processing techniques such as the spin-coating method have been used successfully to reveal excellent properties of organic–inorganic halide perovskites (OHPs) for optoelectronic devices such as solar cell and light-emitting diode, but it is essential to explore other deposition techniques compatible with large-scale production. Single-source flash evaporation technique, in which a single source of materials of interest is rapidly heated to be deposited in a few seconds, is one of the candidate techniques for large-scale thin film deposition of OHPs. In this work, we investigated the reliability and controllability of the single-source flash evaporation technique for methylammonium lead iodide (MAPbI_3_) perovskite. In-depth statistical analysis was employed to demonstrate that the MAPbI_3_ films prepared via the flash evaporation have an ultrasmooth surface and uniform thickness throughout the 4-inch wafer scale. We also show that the thickness and grain size of the MAPbI_3_ film can be controlled by adjusting the amount of the source and number of deposition steps. Finally, the excellent large-area uniformity of the physical properties of the deposited thin films can be transferred to the uniformity in the device performance of MAPbI_3_ photodetectors prepared by flash evaporation which exhibited the responsivity of 51 mA/W and detectivity of 9.55 × 10^10^ Jones.

## Introduction

Organo-metal halide perovskites (OHPs) have come into the spotlight as the power conversion efficiency of solar cell using OHPs has increased dramatically in the past few years^1–7^. Since then, OHPs have demonstrated compliant performance in other optoeletronic devices such as light emitting diodes (LEDs)^8–12^, photodetectors^13,14^, lasers^15^ and phototransistors^16^. Out of various methods studied in the field, solution-processing^2,17,18^, thermal evaporation^19,20^ and chemical vapor deposition^21,22^ have gained the most attention as methods for depositing OHP thin films. Especially, spin-coating is the most commonly used lab-scale deposition method because it is a low-cost and easily accessible process. Although some works have reported remarkable device performances in large-area perovskite optoelectronic devices made with spin-coated perovskite films^9,23^, the solution-process fundamentally imposes limitations in reliably producing uniform films over a large area. In addition, the spin-coating methods have evolved to achieve high-quality OHP films for state-of-the-art devices by adopting additional techniques^17^ such as hot-casting^2,5^, solvent engineering^24,25^ and two-step sequential deposition^3,26,27^, which inevitably adds complexities, and therefore reduces the overall controllability of the process.

The evaporation method, on the other hand, has a potential for uniform large-area film deposition^28,29^, conformal film deposition on uneven surfaces^30^, as well as a simple patterning with shadow masks^31^. Additionally, since it is a solvent-free process, there is no need to consider surface tension or solubility of the underlying layer. Organo-halide precursor (e.g. methylammonium iodide, MAI) and lead-source precursor (e.g. lead iodide, PbI_2_) can be thermally evaporated by co-evaporation method^19,32^, sequential deposition^33–35^ or vapour-assisted deposition^36,37^ to form OHP (e.g. methylammonium lead iodide, MAPbI_3_) films. Although these deposition methods are well-established, it is still challenging to produce OHP films with the desired stoichiometric ratio between the three different ionic components by evaporation because the precursors have very different vaporization temperatures^28^.

Flash evaporation method has gained attention as a candidate for evaporating two or more precursors from a single thermal source by rapidly raising the temperature in a very short time^20,30,31,38–41^. In principle, the rapid vaporization of the precursors induces complete and uniform evaporation of the precursors, while maintaining the same ratio between the different components in OHP. Solar cells with flash evaporated OHP films have exhibited over 10% of power conversion efficiency^39,41^, which is comparable to the early stage spin-coated OHP films^17,42^. Furthermore, the flash evaporation method has been expanded to deposit OHP films with mixed cation and halide species^30^, which is challenging for the aforementioned other evaporation methods^28^. Although this aspect of flash evaporation presents a prospect of exploring a diverse compositional range of OHPs, there has been relatively a few reports which have systematically studied the controllability of the flash evaporation method and the uniformity of OHP films produced by this method. Especially, flash evaporated OHP films have only been reported to be uniform in small areas, but wafer-scale uniformity has rarely been investigated to assess its applicability for mass-producing devices with uniform performance. In this paper, we demonstrate that OHP films with wafer-scale uniformity can be formed by flash evaporation. In addition, it is difficult to monitor the deposition rate and control the resulting film thickness with flash evaporation due to the rapid nature of the evaporation process, unlike other methods. For optoelectronic devices, the thickness of the active layer is critical in determining the device performance^43,44^. Therefore, a reliable deposition of OHP films with controllability over a wide range of target thicknesses is desired for meeting different requirements in terms of film characteristics for various device applications. Our study directly shows that the thickness of flash evaporated OHP films can be controlled by simply adjusting the mass of the source material. Similarly, we discovered that the grain size of the flash evaporated OHP films varied with the mass of the source materials loaded, and that the grain size could even be controlled by introducing multi-step depositions.

## Results and discussion

**Figure 1:**
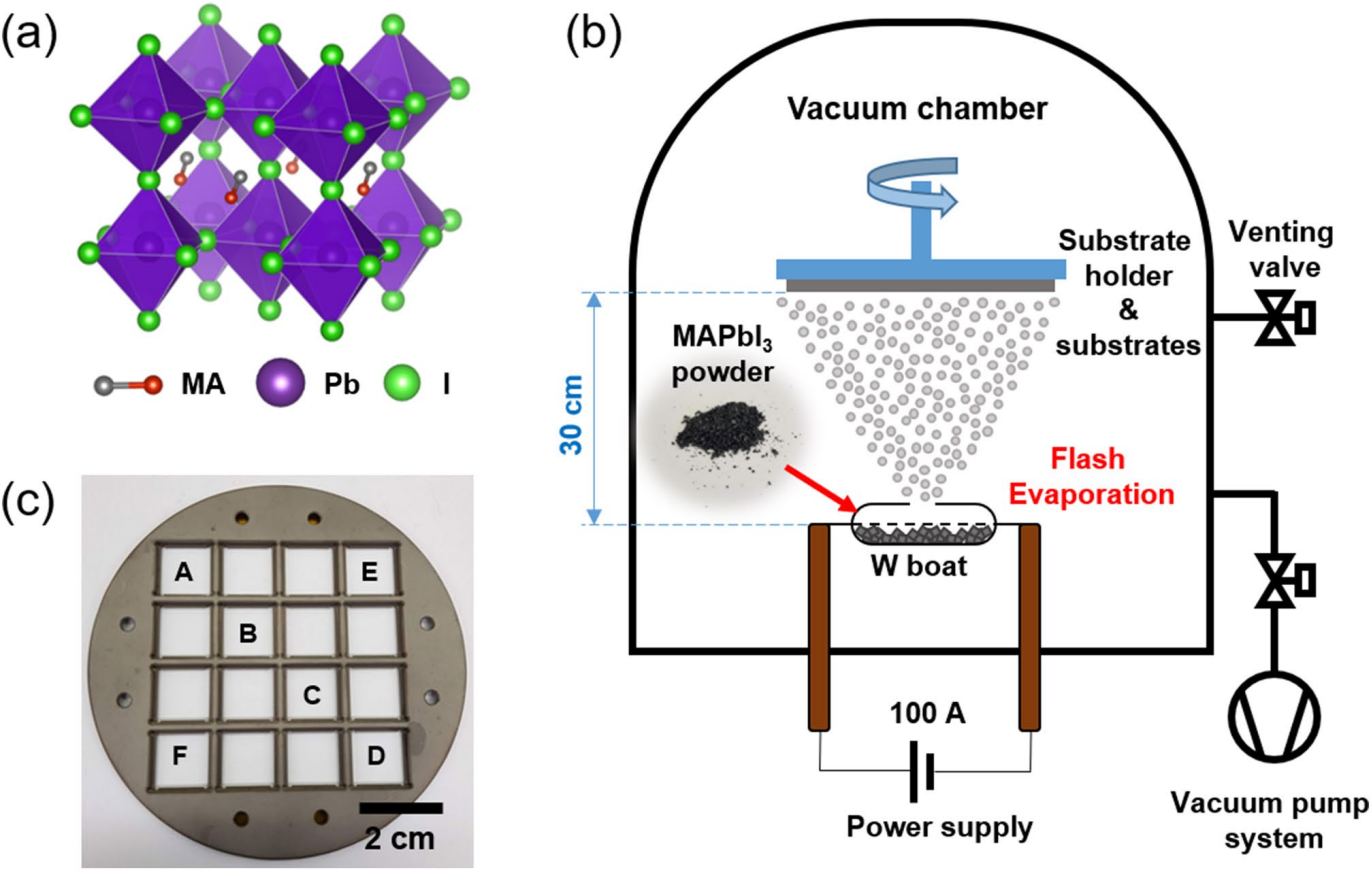
(**a**) Schematic illustration of MAPbI_3_ crystal structure. (**b**) Schematic illustration of deposition of organo-halide perovskite film via flash evaporation. The inset shows photographs of MAPbI_3_ single crystal powder. (**c**) A photograph of the substrate holder for film uniformity test with the labels that indicate the location of the substrates (from A to F).

In this study, we focused on the deposition of MAPbI_3_ films (see Fig. 1a for the crystal structure) by flash evaporation. Figure 1b shows a schematic image of the flash evaporation process adopted in this work. The pre-synthesized MAPbI_3_ single crystal powder was used as the source instead of PbI_2_ and MAI precursors (see the inset of Fig. 1b) in order to obtain better quality films owing to an exact stoichiometric ratio between the different ionic components of MAPbI_3_ within the single crystal^30,45^. The exact amount of single crystal powder was loaded on the tungsten boat which is located inside of vacuum chamber. The source-to-substrate distance was designed to be 30 cm which is the longest distance among source-to-substrate distances of flash evaporation reported so far^20,31,38,40,41^. This is so that we could achieve a uniform deposition of MAPbI_3_ over a large area at the substrate end. The MAPbI_3_ single crystal powder was heated by rapidly ramping up the heater current to 100 A in 3 s at a constant voltage of 0.31 V. The powder was then evaporated within 60 s and deposited on substrates which were located on specific locations of the holder. Throughout this paper, we will refer to different sample locations in the 4-inch wafer size substrate holder as labeled in Fig. 1c (substrate location A to F) to assess the uniformity of the deposited MAPbI_3_ film.

**Figure 2:**
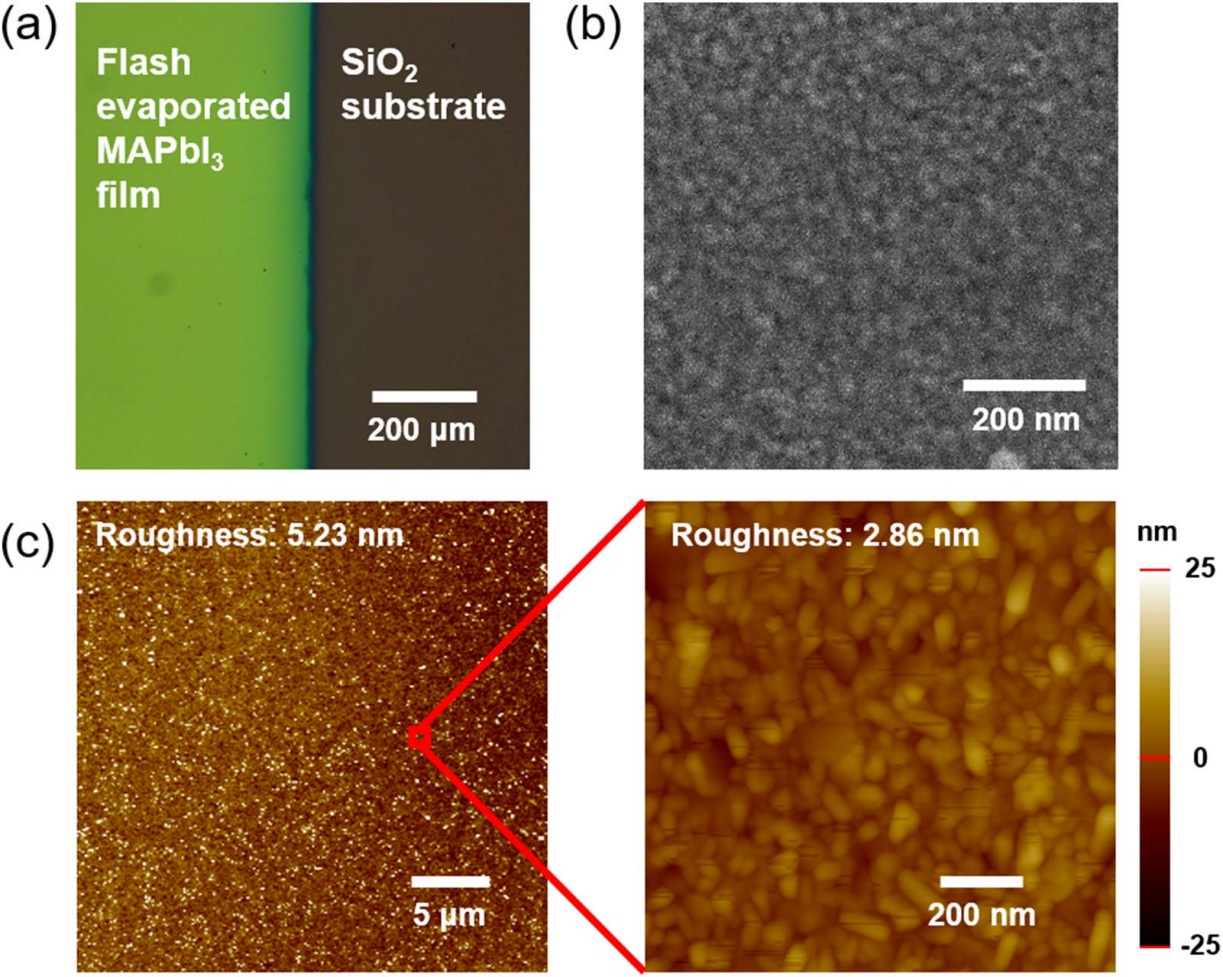
(**a**) An optical microscope image of the flash evaporated MAPbI_3_ film. (**b**) SEM image and (**c**) AFM images of flash evaporated MAPbI_3_ film surface.

**Figure 3:**
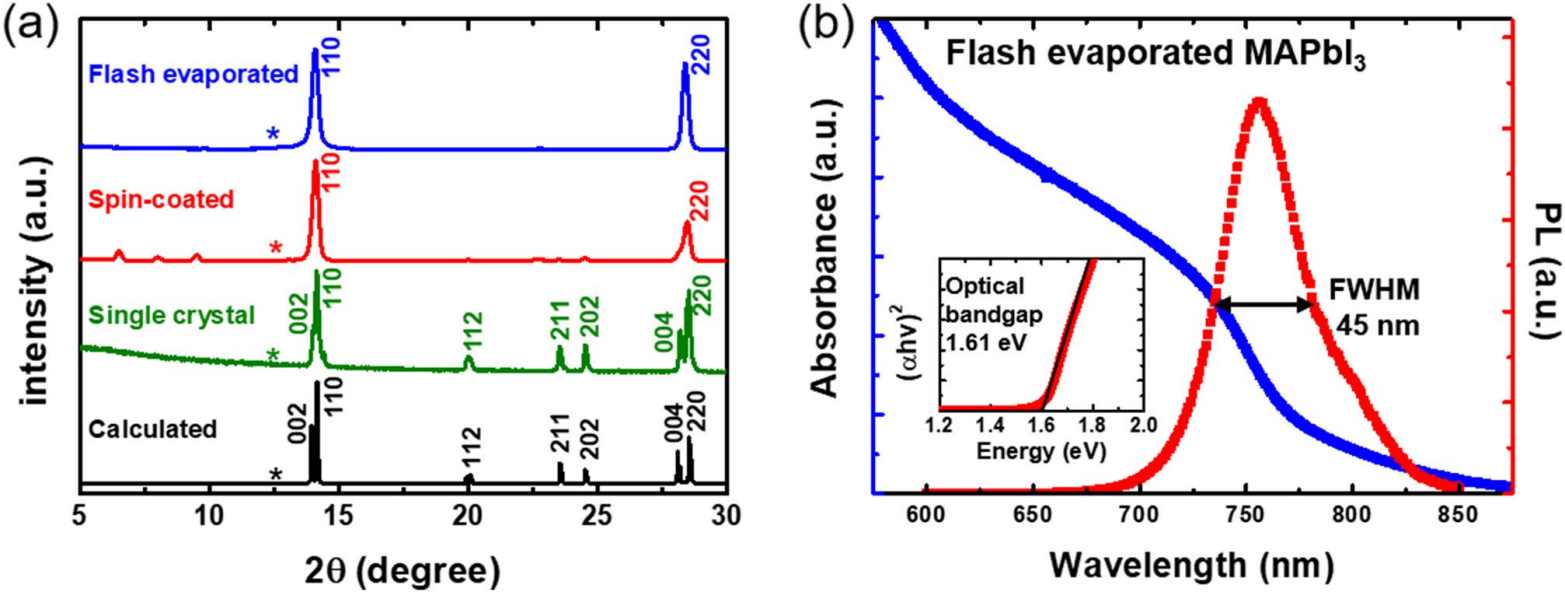
(**a**) XRD data of the flash evaporated film, spin-coated film and single crystal powder. Calculated results from the unit cell of MAPbI_3_ are also shown. (**b**) UV–visible absorbance and PL spectra of MAPbI_3_ film deposited via flash evaporation. The inset shows Tauc plot to estimate the optical bandgap of the perovskite film.

We checked the film quality of flash evaporated MAPbI_3_ films by probing their structural and optical properties as shown in Figs. 2 and 3. An optical micrograph of the flash evaporated MAPbI_3_ film patterned by a shadow mask showed a smooth and clean film with a clearly distinguishable boundary at the edge (see Fig. 2a). The top-surface images of the films measured by field emission scanning electron microscope (FE-SEM) and atomic force microscope (AFM) are presented in Fig. 2b,c, respectively. A typical grain size determined from the FE-SEM image is 40 nm which we will discuss further later in the paper. A smooth and pinhole-free surface was observed with the roughness of approximately 5 nm (2.86 nm locally, Fig. 2c).

Figure 3a shows the X-ray diffraction (XRD) results. The green line shows the XRD result of the single crystal powders of MAPbI_3_ used as the source, which closely resembles the calculated XRD results. It signifies that a high purity MAPbI_3_ single crystal powders were successfully synthesized. The blue and red lines show the XRD results of the flash evaporated and spin-coated MAPbI_3_ films, respectively. The positions of the (110) and (220) peaks were the same for all the XRD results (14.1° and 28.5°, respectively), confirming the identical crystal structure of the flash evaporated MAPbI_3_ film with those prepared by other methods. As no peaks other than (110) and (220) peaks appeared, the deposited MAPbI_3_ films exhibit a strong preferred orientation along the (110) surface^30,32,46,47^. In addition, the high purity of the flash evaporated film is indicated by the absence of diffraction peaks that correspond to PbI_2_ (asterisk marks (12.6°)). Note that this is an interesting observation because many previous studies^31,38,40,41^ have demonstrated that the addition of excess MAI was necessary to deposit pure MAPbI_3_ films without PbI_2_ impurities (detailed discussion could be found in the Supplementary Information Sect. 1).

UV–visible absorbance and photoluminescence (PL) spectra were taken to investigate the optical properties of the flash evaporated MAPbI_3_ film (see Fig. 3b). The estimated optical bandgap from the absorbance spectrum by using the Tauc plot^48^ is 1.61 eV (see the inset of Fig. 3b) and PL peak is shown at 756 nm with a full-width-half-maximum (FWHM) of 45 nm, both of which agree well with the reported values for MAPbI_3_ in literature^15,49^. When compared with the spin-coated MAPbI_3_ film produced as a reference sample, it showed similar absorbance and PL spectra (see Fig. S1 in the Supplementary Information). From the structural and optical characterizations, we could safely confirm that our flash evaporated MAPbI_3_ films had a high film quality without a significant amount of impurities formed.

We checked that the evaporated perovskite films had a uniform thickness and the same optical properties over the whole wafer. Before testing wafer-scale film uniformity, we compared the film uniformity between the flash evaporated perovskite film to spin-coated perovskite film (reference) on the 1.5 × 1.5 cm^2^ substrate. The thickness values of both films were measured by randomly selecting 20 points on cross-sectional FE-SEM images (see Fig. S2 in the Supplementary Information). The average thickness values of the flash evaporated and spin-coated films were similar (207.1 nm and 225.0 nm, respectively), while the standard deviation for the spin-coated film was about 10 times larger (30.2 nm compared to 3.0 nm for the flash evaporated film). Given that the standard deviation value of 3.0 nm for the flash evaporated film is similar to the surface roughness value measured by AFM, the variation in the sampled thickness values can be assumed to be due to the morphology, not the variation in the actual thickness within the film. It can be seen that the film made by flash evaporation has a much uniform thickness and a smooth surface.

**Figure 4:**
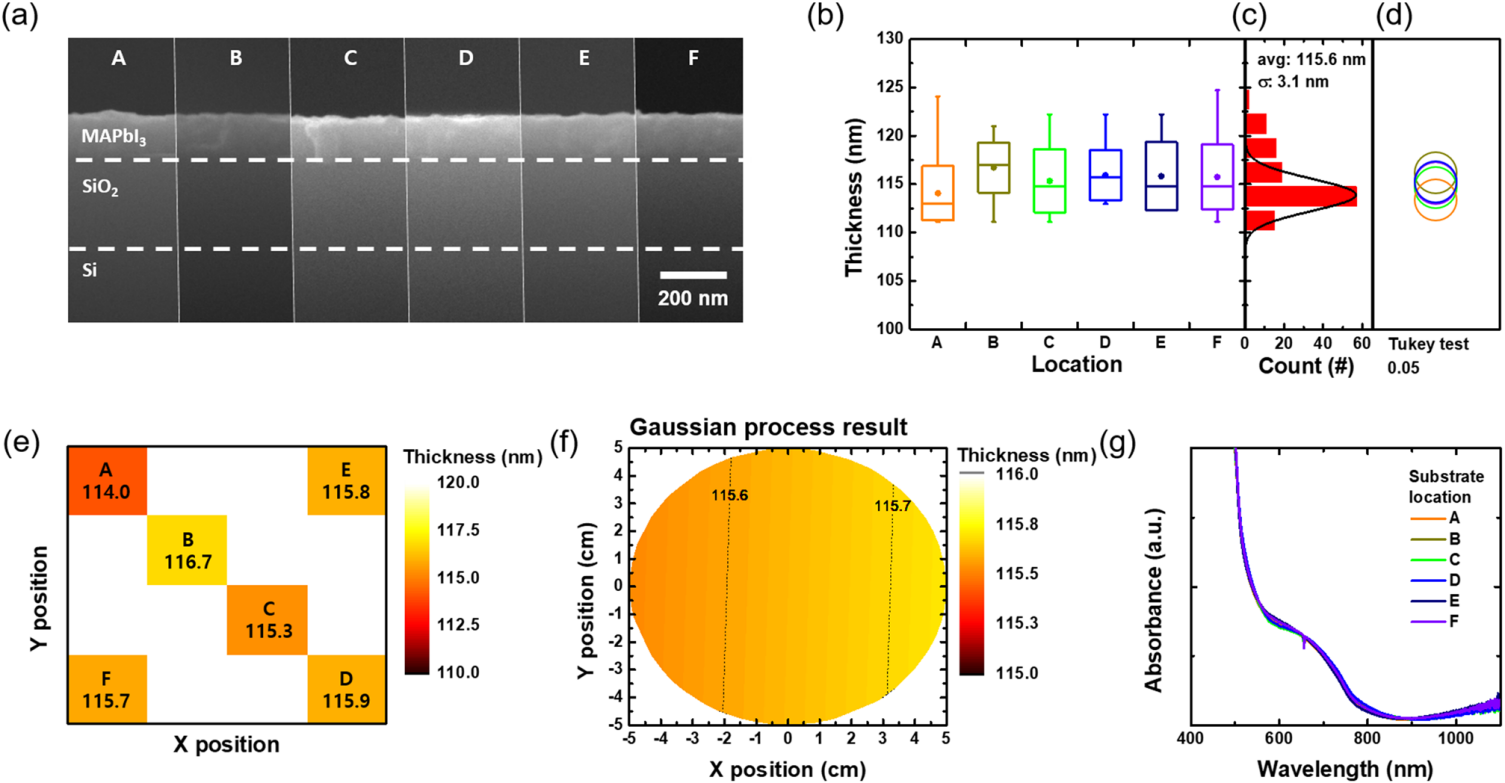
Uniformity test of flash evaporated MAPbI_3_ films. (**a**) Cross-sectional SEM images for the thickness comparison of the MAPbI_3_ film by the substrate location given in Fig. 1c. (**b**) The measured thickness values presented in box and whisker diagram at each location. (**c**) A histogram of all the thickness data. (**d**) Comparison circles from the Tukey test. (**e**) Color map image of the average thickness values at each substrate location on the 4-inch wafer. (**f**) The estimated thickness of the perovskite film by Gaussian process. (**g**) UV–visible absorbance spectra of the MAPbI_3_ films at the different substrate locations.

In order to investigate whether there was a change in the thickness depending on the location over the 4-inch wafer, cross-sectional FE-SEM images were taken for the evaporated films at each substrate location labeled according to Fig. 1c (Fig. 4a). The thickness values were measured at 20 points of the film for each substrate in order to carry out statistical analysis. Figure 4b is a graph summarizing the thickness values extracted from each substrate location drawn as a box and whisker diagram. The dots within the boxes represent the average values and boxes show the first and third quartile range of each distribution. The lines inside the box represent median values and the whiskers show the minimum and maximum values. The box and whisker diagrams show the similarity in the distribution of the thickness values at different locations. Figure 4c shows the distribution for all the measured 120 thickness values from the different locations shown in Fig. 4b plotted together in one histogram. The thickness values did not significantly deviate from the average value of 115.6 nm (the standard deviation was 3.1 nm) at all substrate locations. More importantly, there were no multiple peaks in the normal distribution fit, which suggests that all the thickness values belong to a single distribution. Tukey–Kramer honest significant difference test (Tukey test)^50^ was performed to quantitatively determine whether the distributions of the thickness values at the six different substrate locations (shown in Fig. 4b) can be judged as the same distribution. Tukey test is a statistical test that compares multiple distributions simultaneously and shows how different they are from each other, which can be used to categorize similar distributions into separate groups. The detailed descriptions and raw data are presented in Sect. 4 in the Supplementary Information. Figure 4d is a graphical visualization of the Tukey test results. The comparison circles are shown in Fig. 4d have their centers each aligned with the average thickness values and the radii proportional to the standard deviation values of each distribution. The more the comparison circles overlap, the more similar the distributions are. Here, the comparison circles are all overlapped and therefore all the distributions can be judged as the same distribution sampled from the same population. Analysis of variance (ANOVA) test^51^ was also run to support whether the average values of two or more distributions are statistically identical (see Sect. 4 in Supplementary Information). Thus, all the average thickness values at each substrate location can be considered statistically identical. To visualize the uniformity in the film thickness over the whole 4-inch wafer, we used a color map to plot the average values of the film thickness at each substrate location from A to F (Fig. 4e). The average thickness values at each substrate location differed by less than 2 nm which is smaller than the standard deviation value of 3.1 nm (Fig. 4c). Figure 4f shows simulation results obtained by the Gaussian process regression with the whole 120 thickness data. The variation of the predicted thickness across the wafer was as small as approximately 2 Å. In addition to the thickness measurement, UV–visible absorbance and PL spectra were measured for the films deposited at each substrate location to confirm that they all have the same absorbance and PL responses regardless of location (see Fig. 4g and Fig. S3 in the Supplementary Information). All these results consistently support the wafer-scale uniformity of the flash evaporated perovskite film over the 4-inch wafer.

**Figure 5:**
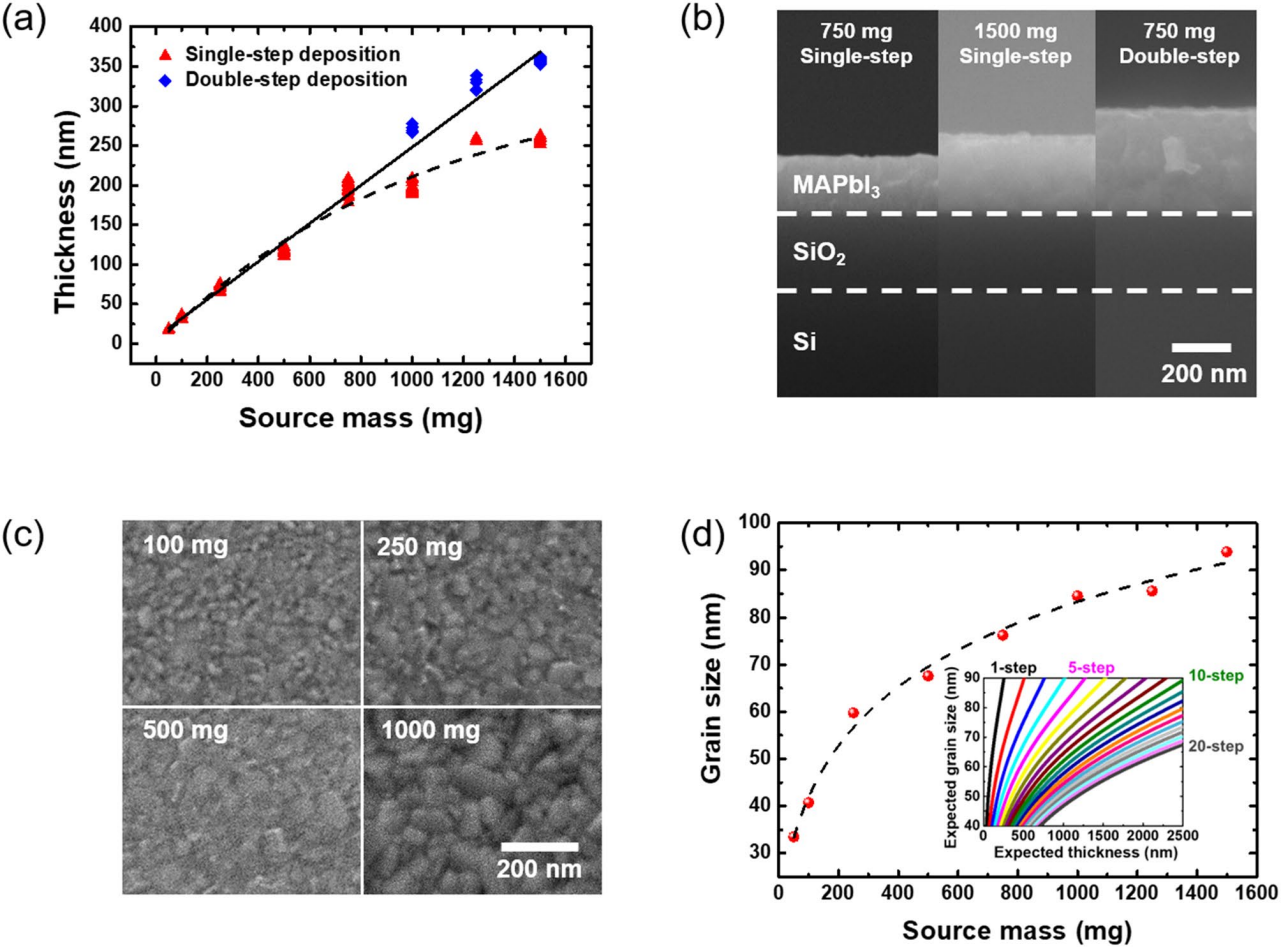
(**a**) A graph of thickness of the flash evaporated perovskite film as a function of the amount of the MAPbI_3_ single crystal power source. (**b**) Cross-sectional SEM images for a single- and multi-step deposited perovskite films by flash evaporation for comparison. (**c**) Top-view SEM images for showing grain size variation for deposition with different source mass. (**d**) Grain size correlation graph of the deposited perovskite films according to the source mass. The inset shows a predicted controllable range of the grain size and thickness of the MAPbI_3_ films by the empirical fit shown as the dashed line.

The controllability of the flash evaporation method was demonstrated by depositing various thicknesses of perovskite films by varying the weight of the source materials. The thicknesses of the films were measured by using a cross-sectional FE-SEM as in the uniformity measurement. The thickness increased linearly with increasing the weight of the source from 50 to 750 mg (see the red triangle points in Fig. 5a). However, as the weight of the source exceeded 750 mg, the increase in the thickness became sub-linear. In order to mitigate the non-linear relationship above the threshold weight of the source of 750 mg, we introduced a multi-step deposition (i.e. the perovskite films were successively deposited multiple times). For example, to deposit a target thickness of 250 nm, 500 mg of the source perovskite powders were deposited twice (a total of 1000 mg), which could then be described by a linear relationship again (see the blue diamond points in Fig. 5a). Figure 5b shows the representative cross-sectional SEM images of MAPbI_3_ films deposited with different weights of the source. Flash evaporation with 1500 mg of the source powders does not yield twice the thickness of the MAPbI_3_ film with 750 mg of the source powders. However, successively evaporating 750 mg of the source twice gives a MAPbI_3_ film twice the thickness (See Fig. 5b).

We discovered that the grain size could also be controlled by varying the weight of the source powders. The grain size tended to increase as the source mass increased (Fig. 5c,d). We also discovered that the grain size did not vary significantly depending on the number of deposition steps while the thickness increased linearly for a double-step (390 nm) and triple-step (620 nm) evaporated films for the source mass of 750 mg (see Fig. S4 in the Supplementary Information for more details), which potentially provides a way for controlling the grain size independently with the thickness (see the inset of Fig. 4d for the predicted range of grain size for each thickness). The grain size of crystals in perovskite films, along with its thickness, is an important parameter that determines the device performance of optoelectronic devices. In the case of solar cells, the carriers should be able to move freely from the active layer (the point of generation within) to the electrodes (where they are extracted), so the larger the grain, the better the collection efficiency^40^. In the case of LEDs, a higher rate of recombination is desired, and therefore a smaller grain size would be required to fabricate LEDs with higher emission efficiencies^52^. Therefore, our findings can be highly relevant for investigating the relationship between the grain size and device performance of optoelectronic devices based on flash evaporated perovskite films.

**Figure 6:**
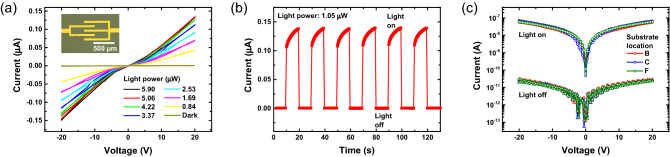
Device characteristics of photodetectors prepared by flash evaporation. (**a**) I–V characteristics under 520 nm laser with different intensities. The inset shows the optical microscope image of the fabricated MAPbI_3_ photodetector. (**b**) Time-dependent photoresponse of the photodetector under few cycles of turn-on and off. (**c**) The I–V characteristics under light and dark conditions for the photodetectors prepared by the flash evaporated films at the different substrate locations.

In order to demonstrate how the wafer-scale film uniformity discussed so far can be transferred to the uniformity in the optoelectronic device performance, we fabricated photodetectors which are one of the most suitable devices due to their simple structures that require only the deposition of two top contact electrodes on evaporated perovskite films (see the inset of Fig. 6a for the device structure). For performance comparison, a photodetector using spin-coated MAPbI_3_ film was also fabricated. The data for the photodetector with spin-coated MAPbI_3_ film are shown in Figs. S5 and S6 in the Supplementary Information. The detailed fabrication process is explained in the Methods section. Figure 6a shows typical current–voltage curves of the photodetector with the evaporated film under light illumination with 532 nm wavelength and various laser intensities. The photocurrent gradually increased with increasing the laser intensity due to increased photogenerated carrier concentrations (see Fig. S6(a) in the Supplementary Information). The responsivity (R) which is the ratio of the excess current generated by light illumination to the incident light power was studied. The responsivity decreased as the light power increased (see Fig. S6(b) in the Supplementary Information). This can be attributed to the increase of carrier–carrier scattering or filling the deep trap states with a longer lifetime, which tends to provide a higher photocurrent at a lower light power^53–55^. The estimated responsivity is 51 mA/W for the photodetector with the flash evaporated film and 137 mA/W for the photodetector with the spin-coated film at a bias of 20 V and light power of 0.84 μW. Detectivity (D*) which is another parameter to characterize the sensitivity of photodetection was calculated according to $D^{*} = R\left( {\frac{{2eI_{dark} }}{A}} \right)^{{ - \frac{1}{2}}}$, where I_dark_ is the dark current, A is the area of the photosensitive region and e is the electric charge (see Fig. S6(c) in the Supplementary Information). The highest value of detectivity was found to be 9.55 × 10^10^ Jones within the measured range for the photodetector with the flash evaporated film. This is a comparable value to the detectivity of 1.53 × 10^11^ Jones for the device with the spin-coated film. This is a comparable value to the detectivity of 6.14 × 10^11^ Jones for the device with the spin-coated film. These device performance parameters are comparable to the previously reported MAPbI_3_-based photodetectors^31,56–58^ and commercial Si photodetectors (< 0.2 A/W)^47,59^. Figure 6b displays repeated on/off operation of the photodetector with the flash evaporated MAPbI_3_ film. The device showed relatively fast photo-responses (< 1 s), stable and reproducible operation during the measurement cycles. Finally, in order to demonstrate how the wafer-scale film uniformity discussed above can be transferred to the uniformity in the photodetector device performance, we fabricated photodetectors with flash evaporated films at different locations (see Fig. 6c). The measured device characteristics were nearly identical regardless of the sample substrate locations (B, C, and F), which shows that we can achieve the wafer-scale uniformity in the device performance by our flash evaporation method.

## Conclusions

We designed a single-source flash evaporation setup with a long source-to-substrate distance to deposit MAPbI_3_ films directly over 4-inch wafer. The thicknesses of the films were measured at various locations of the 4-inch wafer and statistically analyzed to demonstrate that the thicknesses of the films were constant throughout the whole 4-inch wafer. The optical properties of the flash evaporated films were also identical throughout the wafer. The correlation between the amount of the single crystal perovskite powders loaded to the source and the thickness of the deposited film was studied to demonstrate the controllability of the evaporation. We observed that the deposited MAPbI_3_ film thickness was proportional to the source mass until a critical point, above which the film thickness started to saturate. The proportionality was recovered by introducing the multiple numbers of deposition steps which additionally provided a way for controlling the grain size by varying the source mass and number of deposition steps. The wafer-scale uniformity was preserved for photodetector devices fabricated with flash evaporated MAPbI_3_ films. The fabricated devices showed the responsivity of 51 mA/W and detectivity of 9.55 × 10^10^ Jones which are comparable to the previously reported MAPbI_3_-based photodetectors. Our results demonstrate that single-source flash evaporation can be a promising route towards controllably and reliably depositing large-area perovskite films, and therefore producing perovskite-based optoelectronic devices in large-scale.

## Methods

### Synthesis of MAPbI_3_

2.66 g of PbO and 1.90 g of CH_3_NH_3_I (MAI) were dispersed in a mixed acid solution of HI (18 ml, 57 wt% in water) and H_3_PO_2_ (2 ml, 50 wt% in water). The solution was heated at 130 °C until all the precursors were dissolved. The solution was then cooled to room temperature to precipitate MAPbI_3_ single crystals. The crystals were isolated by filtration and dried in vacuum conditions.

### Film preparation

#### Substrate cleaning

The thermally grown 270 nm thick SiO_2_ on Si substrate and glass were sequentially cleaned with acetone, 2-propanol, and deionized water in a sonicator for 10 min at each step. SiO_2_ and glass substrates were exposed to 50 W, 30 sccm condition of O_2_ plasma for 120 s.

#### Deposition of MAPbI_*3*_ film by flash evaporation

Prepared MAPbI_3_ powder was placed into a tungsten boat. After the pressure in a chamber pumped down to below 1 × 10^−6^ Torr, the substrate holder was rotated in 24 rpm for film uniformity, and the current of tungsten boat was rapidly increased to 100 A in 3 s. Then, the temperature of the tungsten boat was raised rapidly and MAPbI_3_ powder sublimated. The nominal deposition rate read by the sensor was approximately 50–80 Å/s. When the deposition rate decreased to 0.1 Å/s, the process was terminated and the total deposition time was within 60 s.

#### Deposition of MAPbI_*3*_ film by spin-coating

Spin-coating was conducted according to the known hot-casting method^2^. 0.5 M of perovskite precursor solution was prepared by dissolving the prepared MAPbI_3_ powder in DMF. The cleaned substrate was heated at 120 °C on the hot plate. Then, the heated substrate was quickly moved to the spin-coater and the precursor solution was spin-coated on the substrate for 40 s at 5000 rpm.

### Fabrication of photodetector

The Au top electrode lines with 50 μm width and 50 nm thickness were deposited using a patterned shadow mask on prepared perovskite film. The electron-beam evaporator pressure was 1 × 10^−6^ Torr and the value of the Au deposition rate on the sensor was approximately 1 Å/s.

### Film characterization

#### SEM measurements

The thickness and surface morphology of the perovskite film were analyzed by FE-SEM (JSM-7800F Prime) using an electron beam accelerated at 5 kV for surface morphology study and 10 kV for thickness study.

#### XRD measurements

Crystallographic structures of perovskite films were analyzed by high resolution X-ray diffraction (HRXRD) technique (Rigaku Smartlab).

#### Steady-state PL measurements

Steady-state PL spectra of the thin film samples (glass/MAPbI_3_ film) were measured using a spectrofluorometer (JASCO FP-8500). The excitation wavelength was 520 nm and used Xenon arc lamp (150 W).

#### Absorbance measurements

The absorbance of the thin film samples (glass/MAPbI_3_ film) was measured using a UV/Vis spectrophotometer (PerkinElmer LAMBDA 45).

#### AFM measurements

Characterization of the perovskite layer surface was performed by an atomic force microscope system (NX 10 AFM, Park Systems).

### Device measurement

The photodetector characteristics of the devices were measured using a semiconductor parameter analyzer (Keithley 4200 SCS) and a probe station system (JANIS Model ST-500). All the measurements were performed in a vacuum environment.

### Data analysis

All data analyzed by the statistical analysis program (JMP software).

## Supplementary information

Supplementary Information
