# Author Correction: Controllable deposition of organic metal halide perovskite films with wafer-scale uniformity by single source flash evaporation

**DOI:** 10.1038/s41598-021-85730-4

**Published:** 2021-03-26

**Authors:** Woocheol Lee, Jonghoon Lee, Hyeon-Dong Lee, Junwoo Kim, Heebeom Ahn, Youngrok Kim, Daekyoung Yoo, Jeongjae Lee, Tae-Woo Lee, Keehoon Kang, Takhee Lee

**Affiliations:** 1 Department of Physics and Astronomy, and Institute of Applied Physics, Seoul National University, Seoul, 08826 Korea; 2 Department of Materials Science and Engineering, Seoul National University, Seoul, 08826 Korea; 3 School of Earth and Environmental Sciences, Seoul National University, Seoul, 08826 Korea; 4 School of Chemical and Biological Engineering, Institute of Engineering Research, Research Institute of Advanced Materials, Nano Systems Institute (NSI), Seoul National University, Seoul, 08826 Korea

Correction to: *Scientific Reports* 10.1038/s41598-020-75764-5, published online 02 November 2020

The original version of this Article contained errors, where values of light power were incorrect due to miscalculation. The light power values were underestimated by 4 times, where the correct values of light power are 4 times larger than the stated values of light power. As a result, also the responsivity and detectivity values contained errors. Specifically, the intensity of light was calculated by dividing the total light power of the laser by the beam size of the incident light, where accidentally the diameter instead of the radius to calculate the beam size was used.

As a result, in the Abstract,

“Finally, the excellent large-area uniformity of the physical properties of the deposited thin films can be transferred to the uniformity in the device performance of MAPbI_3_ photodetectors prepared by flash evaporation which exhibited the responsivity of 0.2 A/W and detectivity of 3.82 × 10^11^ Jones.”

now reads:

“Finally, the excellent large-area uniformity of the physical properties of the deposited thin films can be transferred to the uniformity in the device performance of MAPbI_3_ photodetectors prepared by flash evaporation which exhibited the responsivity of 51 mA/W and detectivity of 9.55 × 10^10^ Jones.”

In the Results section,

“The estimated responsivity is 0.20 A/W for the photodetector with the flash evaporated film and 0.55 A/W for the photodetector with the spin-coated film at a bias of 20 V and light power of 0.21 uW.”

now reads:

“The estimated responsivity is 51 mA/W for the photodetector with the flash evaporated film and 137 mA/W for the photodetector with the spin-coated film at a bias of 20 V and light power of 0.84 μW.”

Additionally,

“The highest value of detectivity was found to be 3.82 × 10^11^ Jones within the measured range for the photodetector with the flash evaporated film. This is a comparable value to the detectivity of 6.14 × 10^11^ Jones for the device with the spin-coated film.”

now reads:

“The highest value of detectivity was found to be 9.55 × 10^10^ Jones within the measured range for the photodetector with the flash evaporated film. This is a comparable value to the detectivity of 1.53 × 10^11^ Jones for the device with the spin-coated film.”

In the Conclusion section,

“The fabricated devices showed the responsivity of 0.2 A/W and detectivity of 3.82 × 10^11^ Jones which are comparable to the previously reported MAPbI_3_-based photodetectors.”

now reads:

“The fabricated devices showed the responsivity of 51 mA/W and detectivity of 9.55 × 10^10^ Jones which are comparable to the previously reported MAPbI_3_-based photodetectors.”

Consequently, Figure 6 of the main article, as well as Figure S5 and Figure S6 of the Supplementary Information file that accompanies this Article, contained errors, where the light power was displayed incorrectly. The original Figure 6 appears below as Figure 1; the original Figures S5 and S6 appear below as Figures 2 and 3, respectively.

**Figure 1:**
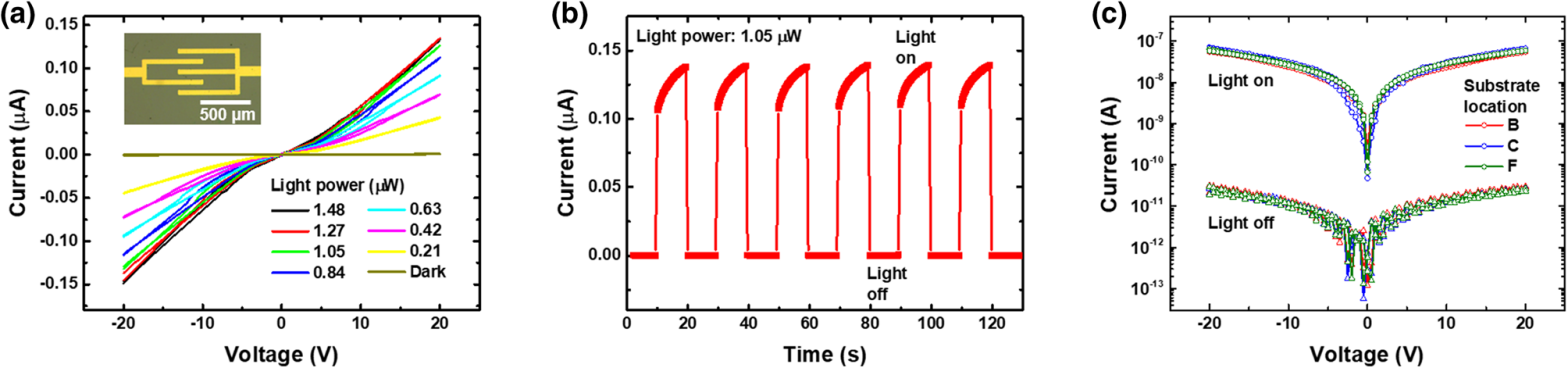
**The original incorrect version of Figure 6**

**Figure 2:**
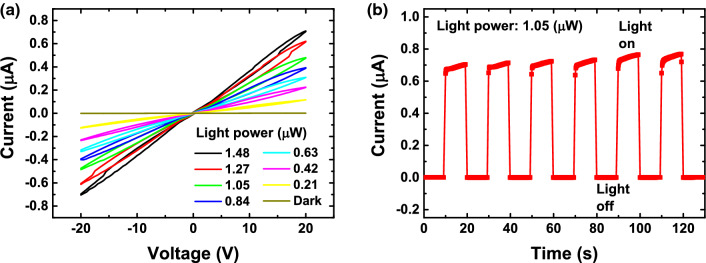
**The original incorrect version of Figure S5**

**Figure 3:**
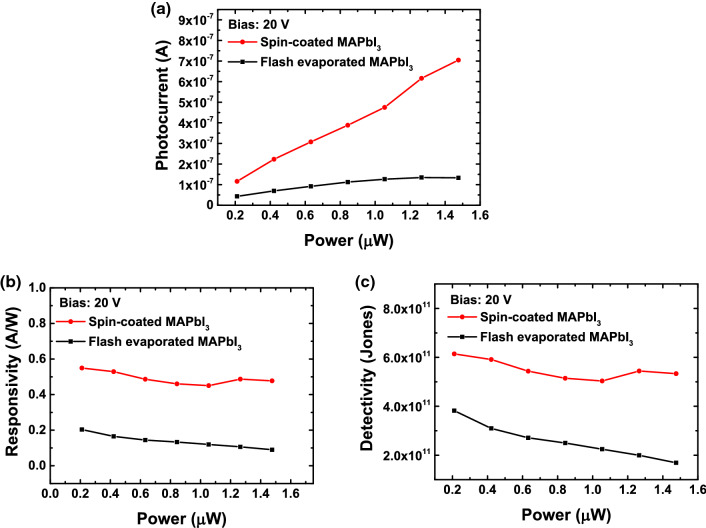
**The original incorrect version of Figure S6**

These errors have now been corrected in the PDF and HTML versions of the Article, and in the Supplementary Information file that accompanies the Article.

