# Supplementary material for: Controllable deposition of organic metal halide perovskite films with wafer-scale uniformity by single source flash evaporation
Source: Sci Rep. 2020 Nov 2;10:18781. doi: 10.1038/s41598-020-75764-5 (PMC7608649; doi:10.1038/s41598-020-75764-5)
Supplement: Supplementary file 1 — Supplementary Information [file 41598_2020_75764_MOESM1_ESM.docx]

Supplementary Information

**Controllable deposition of organic metal halide perovskite films with wafer-scale uniformity by single source flash evaporation**

Woocheol Lee^1,+^, Jonghoon Lee^1,+^, Hyeon-Dong Lee^2^, Junwoo Kim^1^, Heebeom Ahn^1^, Youngrok Kim^1^, Daekyoung Yoo^1^, Jeongjae Lee^3^, Tae-Woo Lee^2,4^, Keehoon Kang^1,^* & Takhee Lee^1,^*

^1^Department of Physics and Astronomy, and Institute of Applied Physics, Seoul National University, Seoul 08826, Korea.

^2^Department of Materials science and Engineering, Seoul National University, Seoul 08826, Korea.

^3^School of Earth and Environmental Sciences, Seoul National University, Seoul, 08826, Korea.

^4^School of Chemical and Biological Engineering, Institute of Engineering Research, Research Institute of Advanced Materials, Nano Systems Institute (NSI), Seoul National University, Seoul 08826, Korea

^+^these authors contributed equally to this work

Correspondence and requests for materials should be addressed to K.K. (email: keehoon.kang@snu.ac.kr) or T.L. (email: tlee@snu.ac.kr)

**1. Experimental conditions and environments for MAPbI_3_ flash evaporation**

Many papers have reported the use of excess MAI to reduce the presence of PbI_2_ in deposited MAPbI_3_ films ^S1-4^. However, each study used a different optimized excess MAI ratio to lower the ratio of PbI_2_ in the deposited film. P. Fan et al. ^S5^ reported that MAPbI_3_ films without PbI_2_ were formed with no excess MAI used in the source, like in our study. This suggests that it is difficult to directly compare the results of the film purity from different studies because each study has different experimental conditions. The following table summarizes the factors that are considered to affect MAPbI_3_ purity.

**Table S1.** Experimental conditions for MAPbI_3_ flash evaporation

|  | MAI:PbI_2_ | Source preparation | Source holder | Distance from source to substrate | Heating method |
| --- | --- | --- | --- | --- | --- |
| M. Tai *et al.*^S1^ | 1.5 : 1 | Spin-coated MAPbI_3_ | CNT sheets | 1 mm | Laser heating |
| H. Wei *et al.*^S2^ | 2.2 : 1 | Spin-coated MAPbI_3_ | CNT sheets | 1 mm | Laser heating |
| H. Xu *et al.*^S3^ | 2 : 1 | Blade coated MAPbI_3_ | Molybdenum foil | 5 cm | Joule heating |
| G. Longo *et al.*^S4^ | 3 : 1 | Blade coated MAPbI_3_ | Tantalum foil | 10 cm | Joule heating |
| P. Fan *et al.*^S5^ | 1 : 1 | MAPbI_3_ single crystal powder | Crucible | Not mentioned | Joule heating |
| This work | 1 : 1 | MAPbI_3_ single crystal powder | Tungsten boat | 30 cm | Joule heating |

**2. UV-visible absorbance and PL spectra of spin-coated MAPbI_3_ film**

**Figure S1.**  UV-visible absorbance and PL spectra of spin-coated MAPbI_3_ film.

**3. MAPbI_3_ film thickness measurement using SEM**

The images of cross-section SEM were taken at various locations and a total of 20 thickness values were measured at a single substrate location (see Fig. S2(a)). Figure S2(b) is a distribution of the measured thicknesses of the flash evaporated MAPbI_3_ film at a single substrate location (see Fig. 1(c) in the main manuscript). The standard deviation (σ) was 3.0 nm. For comparison, the thickness of the MAPbI_3_ film deposited via spin-coating was also measured in the same way (see Figs. S2(c) and S2(d)). The σ of the spin-coated MAPbI_3_ film was found to be 30.2 nm, which shows that the flash evaporated MAPbI_3_ film is much more uniform than the spin-coated MAPbI_3_ film.

**Figure S2.** Examples of thickness measurements using cross-section SEM images of (a) the flash evaporated MAPbI_3_ films and (c) spin-coated MAPbI_3_ film. Thickness distribution of (b) the flash evaporated MAPbI_3_ film and (d) spin-coated MAPbI_3_ film.

**4. Tukey-Krammer HSD test and ANOVA test results report**

Analysis of variance (ANOVA) test can be used to check whether several groups forming a normal distribution can be judged as the same group. This test method verifies that the various groups forming the normal distribution can be regarded as the same group average. In ANNOVA test, the null hypothesis (H0) is that the mean of all groups is the same, and the alternative hypothesis (H1) is that the average of more than one group is different.

In order to extract p-values ​which are the most important values in this method, the degrees of freedom, mean square, and F ratio are required. The degree of freedom of the label is 5 (= (n − 1)) is obtained for the number of 6 (= n) labels. The degree of freedom of the error is given as 114 (= (t − n)) for the result of 120 (= t) film thickness measurements, and the total degree of freedom is 119 (= (t − 1)). The sum of squares of the labels can be obtained by summing the squares of the difference between the average and the six averages of each label. And also, the sum of squares of errors can be obtained by summing all the differences between the mean of a specific group and the group composition. The sum of square divided by each degree of freedom is given as mean square (MS) which is placed at the 4th column in ANOVA table, and the F ratio can be obtained using relationship F ratio = MS_Label_ / MS_Error_. Subsequently, the p-value can be obtained as the position of the F ratio in the F-distribution, and it can be determined whether or not to adopt the null hypothesis according to the p-value. If the reference level is 0.05, which is a commonly used significance level, it can be said that the average of one group is different when it is 0.05 or less. Conversely, when the p-value exceeds 0.05, it can be confirmed that all groups have the same average. As a result of the experiment, it can be seen that all films have the same average with the p-value of 0.147.

The Tukey-Krammer test is a method that can be used to determine whether the group is the same for each group that forms a normal distribution. While the ANOVA test judges whether one unknown group is different, the Tukey-Krammer test can compare each group to find which group is different in detail. The average difference between each group can be confirmed by the difference, and whether each group is a different group can be determined through p-value. When setting the significance level to 0.05, if it is less than 0.05, it can be judged as another group, and it can be confirmed from the table that the larger the difference, the smaller the p-value. A film and B film, which have the most difference, have the most difference with 26.5 Å, but it can be judged as the group having the same average with a p-value of 0.071. The comparison results for each are summarized in the connecting letter report, and when they are all judged to be classification A, it can be reconfirmed that all films form the same cluster.

**Table S2.** The standard information of the ANOVA test.

| **ANOVA test result** | | | | | |
| --- | --- | --- | --- | --- | --- |
| Source | Degree of freedom | Sum of squares | Mean square | F ratio | p-value |
| Label | 5 | 7699.6 | 1539.92 | 1.670 | 0.147 |
| Error | 114 | 105102.3 | 921.95 |  |  |
| Total | 119 | 112801.9 |  |  |  |

**Table S3.** (a) The results of the comparison pairs of the substrate locations from A to F. (b) Classification results of the substrate locations. All the 6 substrate locations were classified to be in the same group.

**5. PL spectra according to the substrate location for uniformity test**

**Figure S3.** PL spectra of the flash evaporated MAPbI_3_ films according to the substrate location. All the spectra showed the same peak position.

**6. Multi-step flash evaporation**

**
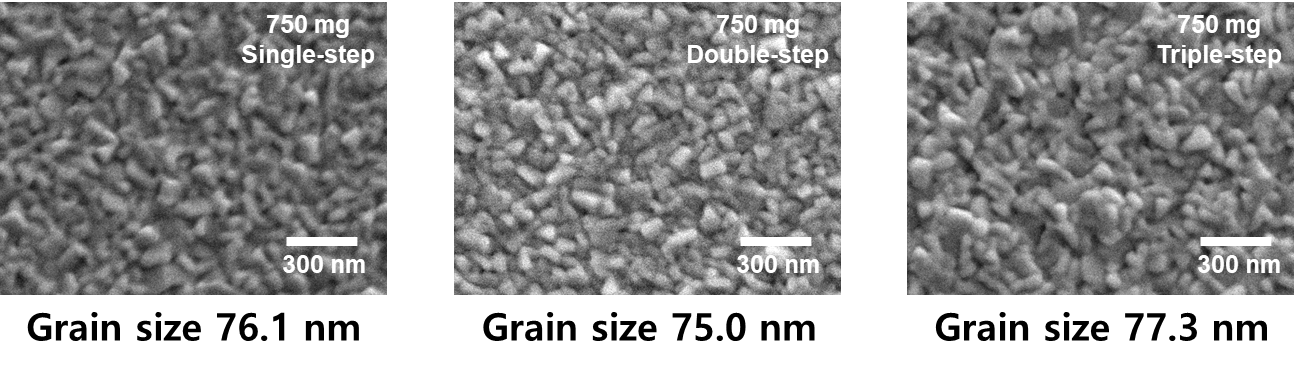
**

**Figure S4.** SEM images of multi-step-deposited flash evaporated MAPbI_3_ film surfaces for single-step, double-step and triple-step deposited MAPbI_3_ films.

**7. Device performance of photodetectors with the spin-coated MAPbI_3_ film**

**Figure S5.** Device characteristics of the photodetectors prepared by spin-coating. (a) I-V characteristics under 520 nm laser under light illumination with different intensities. (b) Time-dependent photoresponse of the spin-coated MAPbI_3_ photodetector under few cycles of turn-on and off.

**8. Photocurrent, responsivity, and detectivity**

**Figure S6.** (a) Photocurrent, (b) photoresponsivity and (c) detectivity of the flash evaporated and the spin-coated MAPbI_3_ photodetectors operated at a bias voltage of 20 V as a function of the light power.

**References**

S1 Tai, M. *et al.* Laser-Induced Flash-Evaporation Printing CH3NH3PbI3 Thin Films for High-Performance Planar Solar Cells. *ACS Appl. Mater. Interfaces* **10**, 26206-26212, doi:10.1021/acsami.8b05918 (2018).

S2 Wei, H. *et al.* Perovskite photodetectors prepared by flash evaporation printing. *RSC Adv.* **7**, 34795-34800, doi:10.1039/C7RA04061J (2017).

S3 Xu, H. *et al.* Resolving the detrimental interface in co-evaporated MAPbI3 perovskite solar cells by hybrid growth method. *Org. Electron.* **69**, 329-335, doi:https://doi.org/10.1016/j.orgel.2019.03.047 (2019).

S4 Longo, G., Gil-Escrig, L., Degen, M. J., Sessolo, M. & Bolink, H. J. Perovskite solar cells prepared by flash evaporation. *Chem. Commun.* **51**, 7376-7378 (2015).

S5 Fan, P. *et al.* High-performance perovskite CH3NH3PbI3 thin films for solar cells prepared by single-source physical vapour deposition. *Sci. Rep.* **6**, 29910, doi:10.1038/srep29910 (2016).
